# Serum lipid and lipoprotein levels of middle-aged and elderly Chinese men and women in Shandong Province

**DOI:** 10.1186/s12944-019-1000-0

**Published:** 2019-03-04

**Authors:** Meijian Wang, Xinguo Hou, Wenchao Hu, Li Chen, Shihong Chen

**Affiliations:** 1 Department of Endocrinology, Qilu Hospital of Shandong University (Qingdao), 758 Hefei Road, Qingdao, Shandong 266035 People’s Republic of China; 2 Department of Endocrinology of Qilu Hospital and Institute of Endocrinology and Metabolism, Qilu Hospital of Shandong University, 107 Wenhuaxi Road, Ji’nan, Shandong 250012 People’s Republic of China; 3 Department of Endocrinology, The Second Hospital of Shandong University, 247 Beiyuan Street, Ji’nan, Shandong 250033 People’s Republic of China

**Keywords:** Shandong Province, Cross-sectional study, Lipids, Lipoproteins

## Abstract

**Background:**

Cardiovascular and cerebrovascular diseases have become leading causes of death in China as the economy develop and lifestyles change. This study aimed to estimate the relationship of the age, gender, and glucose metabolism with the serum lipid and lipoprotein levels of middle-aged and elderly Chinese men and women in Shandong Province.

**Methods:**

We conducted a cross-sectional study in Shandong Province that included 10,028 adults aged ≥40 years. Fasting serum total, low-density lipoprotein (LDL), high-density lipoprotein (HDL) cholesterol and triglycerides were measured by standard methods.

**Results:**

The estimates of total, LDL, and HDL cholesterol and triglycerides were as follows: 5.35, 3.18, 1.51, and 1.34 mmol/L in the middle-aged and elderly Chinese adult population; 5.14, 3.08, 1.42, and 1.33 mmol/L in male subjects; 5.46, 3.24, 1.56, and 1.34 mmol/L in females; 5.27, 3.11, 1.54, and 1.24 mmol/L in the normal glucose tolerance population, 5.49, 3.27, 1.50, and 1.41 mmol/L in the population with pre-diabetes, and 5.39, 3.23, 1.43, and 1.58 mmol/L in the population with diabetes, respectively. Moreover, 36.92 and 19.10% of the adults had borderline-high and high total cholesterol. The population estimates for borderline-high, high LDL and low HDL cholesterol levels were 25.24, 13.39, and 5.64%, respectively. Meanwhile, borderline high and high triglyceride levels accounted for 16.7 and 17.47% of the population, respectively.

**Conclusions:**

Serum total and LDL cholesterol levels were high in the ≥40 years old population of Shandong Province. Age, gender, glucose metabolism status, body mass index (BMI) and glycosylated hemoglobin (HbA1c) can affect serum lipid and lipoprotein levels.

## Background

With the economic developments and adverse changes in lifestyle (such as a decreased physical activity and high-calorie food intake), cardiovascular and cerebrovascular diseases have become the important cause of death in China [1, 2]. 42.8% of deaths in adults aged ≥40 years were estimated to be attributable to heart disease and stroke in 1991–2000 in a Chinese national prospective cohort study [2]. Elevated serum lipids are one of the most important modifiable risk factors for cardiovascular and cerebrovascular diseases in Western [3–5] and Asian [6, 7] populations. We try to estimate the relationship between age, gender, status of glucose metabolism, and serum lipids and lipoproteins among the Chinese men and women aged ≥40 years of Shandong Province.

## Methods

### Study population

In 2012, we conducted an epidemiological survey of type 2 diabetes in Shandong Province. We chose several communities in the cities of Jinan and Jining in Shandong Province. A total of 10,028 individuals participated in the study. After excluding 273 persons with missing demographic information and lipid data, we covered 9755 adults in the final analysis (3313 men and 6442 women). The present work was one part of the baseline survey from the REACTION study that investigated the association of diabetes and cancer, conducted among 259,657 adults aged ≥40 years in 25 communities across mainland China, from 2011 to 2012 [8–11].

The ethics committee of Qilu Hospital of Shandong University approved the study. Written informed consent was obtained from each participant before data collection.

### Data collection

Data collection was conducted at local community hospital or community medical center in the participants’ residential area. During the clinic visits, trained research staff collected information on demographic characteristics, personal and family medical history, medication history, and lifestyle risk factors according to a standard questionnaire [12]. It also included questions about the diagnosis and treatment of dyslipidemia. Cigarette smoking, alcohol drinking, and physical activities were also recorded. Blood pressure, heart rate, height, body weight, and waist circumference were measured by standard methods [13]. Body mass index (BMI) was calculated as weight in kilograms divided by the square of height in meters. Waist-hip ratio (WHR) was calculated as waist circumference in centimeter divided by the hip circumference in centimeter.

Blood samples were drawn by venipuncture after at least 8 h of overnight fasting to measure serum total, low-density lipoprotein (LDL), and high-density lipoprotein (HDL) cholesterol, triglycerides, fasting plasma glucose (FPG), fasting plasma insulin (FINS), glycosylated hemoglobin (HbA1c), creatinine (Cr), alanine aminotransferase (ALT), and aspartate aminotransferase (AST). Homeostasis model assessment insulin resistance (HOMA-IR) was calculated as FPG multiplied by FINS divided by 22.5. Blood specimens were processed at local medical center and sent to the central laboratories.

Diagnosis of diabetes is according to ADA [14]: FPG ≥7 .0mol/L, 2hPG ≥11.1mmol/L, also include those have been diagnosed diabetes and began to therapy. Pre-diabetes include: impaired fasting glucose (IFG), impaired glucose tolerance (IGT) and IFG accompanied with IGT. IFG defines as FPG 5.6–6 .9mmol/L and 2hPG < 7 .8mmol/L. IGT defines as FPG < 7 .0mmol/L and 2hPG 7.8–11.0mmol/L.

Mean levels of total, LDL, and HDL cholesterol and triglycerides were estimated for the overall population by gender and status of glucose metabolism. Moreover, age-specific mean levels were calculated for men and women. Serum lipids and lipoprotein levels were classified on the basis of the Third Report of the Expert Panel on Detection, Evaluation, and Treatment of High Blood Cholesterol in Adults [13]. The prevalence estimates of total, LDL, and HDL cholesterol categories were calculated for the total population and by gender with the use of the direct method according to the population distribution in China in 2008 [15].

### Statistical analysis

All statistical analyses were conducted using the SPSS 19.0. Normally distributed data were expressed as mean ± standard deviation (^−^*x ± s*). None-normally distribution data were expressed as median and quartile. All *P* values were two-tailed and not adjusted for multiple testing. The differences of characteristics between men and women were compared with unpaired *t*-test or chi-square tests. One-way ANOVA was used to compare the differences of characteristics between different aged groups. Age-specific prevalence of dyslipidemia was also calculated.

## Results

Table 1 shows the demographic and clinical characteristics of the study participants categorized into men, women, and overall population.

**Table 1:** Demographic and clinical characteristics of study participants in Shandong Province

	Overall *N* = 9755	Men *N* = 3313	Women *N* = 6442	*P* values
Age, y	58.58 ± 9.81	60.15 ± 9.98	57.78 ± 9.62	< 0.01
BMI, kg/m^2^	26.25 ± 3.48	26.48 ± 3.25	26.13 ± 3.59	< 0.01
Systolic BP, mm Hg	139.55 ± 21.32	142.06 ± 20.26	138.26 ± 21.73	< 0.01
Diastolic BP, mm Hg	80.15 ± 14.34	83.15 ± 18.63	78.60 ± 11.22	< 0.01
Waist circumference, cm	86.86 ± 10.09	90.54 ± 9.40	84.98 ± 9.92	< 0.01
Waist-hip ratio	0.86 ± 0.06	0.89 ± 0.06	0.84 ± 0.06	< 0.01
Glycosylated hemoglobin, %	6.25 ± 1.25	6.32 ± 1.35	6.21 ± 1.19	< 0.01
Fasting glucose, mmol/L	6.16 ± 1.90	6.40 ± 2.10	6.03 ± 1.78	< 0.01
Fasting plasma insulin, uIU/ml	9.43 ± 6.63	8.88 ± 6.34	9.71 ± 6.76	< 0.01
HOMA-IR	2.67 ± 2.72	2.59 ± 2.45	2.71 ± 2.84	< 0.05
Creatinine, ummol/L	65.19 ± 13.78	74.41 ± 16.05	60.45 ± 9.43	< 0.01
Alanine aminotransferase, U/L	11.00(8.00–15.00)	12.00(9.00–17.00)	10.00(7.00–14.00)	< 0.01
Aspartate aminotransferase, U/L	18.00(16.00–22.00)	19.00(16.00–23.00)	18.00(16.00–21.00)	< 0.01
Total cholesterol, mmol/L	5.35 ± 1.05	5.15 ± 0.98	5.46 ± 1.07	< 0.01
LDL cholesterol, mmol/L	3.18 ± 0.86	3.08 ± 0.81	3.24 ± 0.88	< 0.01
HDL cholesterol, mmol/L	1.51 ± 0.38	1.42 ± 0.38	1.56 ± 0.37	< 0.01
Triglycerides, mmol/L	1.34(0.95–1.92)	1.33(0.96–1.91)	1.34(0.95–1.92)	0.41

Mean values (95% confidence interval [CI]) are shown. *BMI* body mass index, *BP* blood pressure, *LDL* low-density lipoprotein, *HDL* high-density lipoprotein, *HOMA-IR* homeostasis model assessment insulin resistance

*P* values between men and women

### Mean lipid and lipoprotein levels

The mean levels of total, LDL, and HDL cholesterol and triglycerides were 5.35, 3.18, 1.51, and 1.34 mmol/L, respectively, in the overall Shandong Province population aged ≥40 years (Table 1). Generally, these mean levels were slightly higher in women than in men. In addition, the mean levels of total and LDL cholesterol and triglycerides increased with age until 60–64 years, and the mean levels of total cholesterol and triglycerides subsequently decreased (Table 2). Overall, total and LDL cholesterol levels increased continuously over the entire age range in women. Serum total, LDL, and HDL cholesterol and triglycerides levels were linearly correlated with blood pressure. Serum total, and LDL cholesterol levels in normal glucose tolerance (NGT) group, pre-diabetic group, and diabetic group increased first and then decreased, while HDL cholesterol level decreased gradually, and triglycerides level increased gradually (Table 2).

**Table 2:** Mean (95% CI) Level of Serum Total, LDL, HDL Cholesterol, and Triglycerides in Shandong Adults Aged ≥40 years, 2012

	Serum Cholesterol, mmol/L			Serum Triglycerides, mmol/L
	Total	LDL	HDL	
Sex- and age-specific				
Men, age, y				
40–44	4.99 ± 0.93	2.90 ± 0.77	1.40 ± 0.31	1.38(1.02–2.18)
45–49	5.20 ± 0.97	3.00 ± 0.79	1.41 ± 0.40	1.55(1.03–2.29)
50–54	5.08 ± 0.96	3.01 ± 0.73	1.43 ± 0.36	1.34(0.93–1.92)
55–59	5.19 ± 1.04	3.12 ± 0.85	1.42 ± 0.39	1.38(0.99–2.07)
60–64	5.19 ± 0.98	3.13 ± 0.83	1.40 ± 0.36	1.34(1.00–1.86)
65–69	5.19 ± 0.90	3.12 ± 0.81	1.42 ± 0.35	1.26(0.92–1.75)
≥ 70	5.11 ± 0.98	3.10 ± 0.80	1.44 ± 0.40	1.24(0.91–1.75)
P values for linear trend	0.29	< 0.05	0.30	< 0.05
Women, age, y				
40–44	4.74 ± 0.90	2.73 ± 0.73	1.59 ± 0.33	1.00(0.75–1.39)
45–49	5.05 ± 1.05	2.94 ± 0.85	1.61 ± 0.37	1.06(0.76–1.59)
50–54	5.42 ± 1.06	3.20 ± 0.87	1.61 ± 0.36	1.26(0.88–1.75)
55–59	5.54 ± 1.00	3.31 ± 0.82	1.56 ± 0.39	1.41(1.02–2.05)
60–64	5.72 ± 1.03	3.40 ± 0.88	1.51 ± 0.34	1.52(1.07–2.15)
65–69	5.67 ± 0.99	3.42 ± 0.83	1.51 ± 0.36	1.46(1.11–2.05)
≥ 70	5.75 ± 1.07	3.44 ± 0.92	1.53 ± 0.40	1.51(1.11–2.10)
*P* values for linear trend	< 0.01	< 0.01	< 0.05	< 0.01
Status of glucose metabolism				
NGT	5.27 ± 1.01	3.11 ± 0.83	1.54 ± 0.38	1.24(0.89–1.76)
Prediabetes	5.49 ± 1.07	3.27 ± 0.88	1.50 ± 0.35	1.41(1.00–1.98)
Diabetes	5.39 ± 1.09	3.23 ± 0.90	1.43 ± 0.39	1.58(1.11–2.27)
P values for difference	< 0.01	< 0.01	< 0.01	< 0.01
Blood pressure				
NBP	5.06 ± 1.13	3.05 ± 0.87	1.60 ± 0.43	1.10(0.80–1.58)
Prehypertension	5.30 ± 1.00	3.11 ± 0.84	1.53 ± 0.35	1.22(0.88–1.74)
Hypertension	5.48 ± 1.01	3.26 ± 0.85	1.47 ± 0.34	1.36(1.05–2.04)
*P* values for difference	< 0.01	< 0.05	< 0.01	< 0.05

*LDL* low-density lipoprotein, *HDL* high-density lipoprotein, *NGT* normal glucose tolerance, Prediabetes contains: (1) IFG, impaired fasting glucose; (2) IGT, impaired glucose tolerance; (3) IFG accompany with IGT. NBP, normal blood pressure, systolic blood pressure < 120 mmHg and diastolic blood pressure < 80 mmHg (1 mmHg = 0.1 33 kPa); Prehypertension, systolic blood pressure 120–139 mmHg and/or diastolic blood pressure 80–89 mmHg; Hypertension, systolic blood pressure ≥ 140 mmHg or diastolic blood pressure ≥ 90 mmHg

### Prevalence of dyslipidemia

**Fig. 1:**
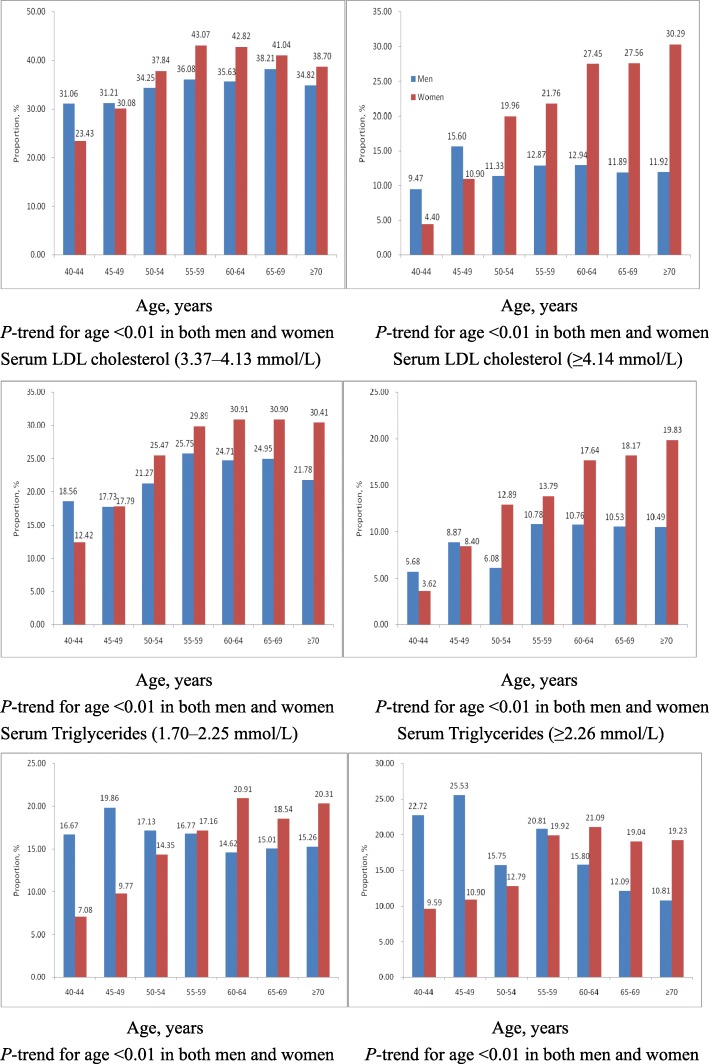
Age-specific proportion of individuals with borderline-high and high total (top) and LDL (middle) cholesterol, as well as borderline-high and high serum triglycerides (bottom), among adults aged ≥40 years in Shandong Province, 2012

**Fig. 2:**
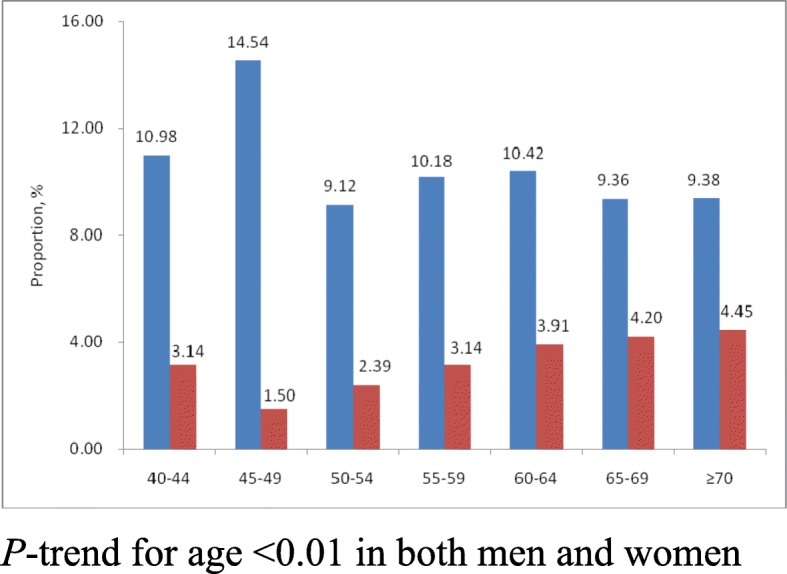
Age-specific proportion of individuals with lower serum HDL cholesterol among adults aged ≥40 years in Shandong Province, 2012

The prevalence of borderline-high (5.18–6.21 mmol/L) and high total cholesterol levels (≥6.22 mmol/L) was 36.92 and 19.10%, respectively (Table 3), and increased with age in both women and men (Fig.1). Meanwhile, the prevalence of borderline-high (3.37–4.13 mmol/L) and high LDL cholesterol (≥4.14 mmol/L) was 25.24 and 13.39%, respectively. The prevalence of borderline-high (1.70–2.25 mmol/L) and high triglycerides (≥2.26 mmol/L) was 16.07 and 17.47%, respectively. The prevalence of low HDL cholesterol (< 1.04 mmol/L) was 5.64% in the overall adult Shandong population aged ≥40 years and increased with age in women (Fig.2).

**Table 3:** Prevalence of Dyslipidemia

	Prevalence, %		
	Overall	Men	Women
Serum total cholesterol, mmol/L			
5.18–6.21	36.92	35.07	37.88
≥ 6.22^a^	19.10	13.40	22.03
Serum LDL cholesterol, mmol/L			
3.37–4.13	25.24	23.03	26.37
≥ 4.14^a^	13.39	10.62	14.81
Serum Triglycerides, mmol/L			
1.70–2.25	16.07	16.12	16.05
≥ 2.26^a^	17.47	17.51	17.45
Serum HDL cholesterol, mmol/L			
≤ 1.04	5.64	10.26	3.26

^a^Includes persons on a lipid-lowering medication

### The correlation of total, LDL, and HDL cholesterol and triglycerides with other clinical characteristics

Simple linear regression analyses showed that age, gender, status of glucose metabolism, BMI, and HbA1c were correlated with total, LDL, and HDL cholesterol and triglycerides (Table 4). Multiple linear regression analyses showed that a) only age, gender, BMI, HbA1c were associated with total lipoprotein; b) only BMI, and HbA1c were associated with triglycerides; c) only gender, status of glucose metabolism, BMI, and HbA1c were associated with HDL cholesterol; and d) age, gender, status of glucose metabolism, BMI, and HbA1c still were associated with LDL (Table 4).

**Table 4:** The Correlation of Total, LDL, and HDL Cholesterol and Triglycerides with Other Clinical Characteristics

	Simple linear regression		Multiple linear regression	
	r	*P*	β	*P*
Serum total cholesterol, mmol/L				
Age, y	0.165	< 0.001	0.169	< 0.001
Gender	0.14	< 0.001	0.163	< 0.001
Status of glucose metabolism	0.074	< 0.001	0.017	0.176
BMI, kg/m^2^	0.042	< 0.001	0.029	0.005
HbA1c, %	0.082	< 0.001	0.045	< 0.001
Serum Triglycerides, mmol/L				
Age, y	0.022	0.03	− 0.018	0.091
Gender	− 0.022	0.026	−0.006	0.553
Status of glucose metabolism	0.125	< 0.001	0.02	0.104
BMI, kg/m^2^	0.138	< 0.001	0.115	< 0.001
HbA1c, %	0.204	< 0.001	0.171	< 0.001
Serum LDL cholesterol, mmol/L				
Age, y	0.168	< 0.001	0.158	< 0.001
Gender	0.086	< 0.001	0.11	< 0.001
Status of glucose metabolism	0.083	< 0.001	−0.042	0.001
BMI, kg/m^2^	0.087	< 0.001	0.066	< 0.001
HbA1c, %	0.163	< 0.001	0.154	< 0.001
Serum HDL cholesterol, mmol/L				
Age, y	−0.067	< 0.001	− 0.02	0.055
Gender	0.176	< 0.001	0.159	< 0.001
Status of glucose metabolism	−0.122	< 0.001	−0.102	< 0.001
BMI, kg/m^2^	−0.257	< 0.001	−0.24	< 0.001
HbA1c, %	−0.055	< 0.001	0.044	< 0.001

*LDL* low-density lipoprotein, *HDL* high-density lipoprotein, *BMI* body mass index

## Discussion

Our study shows that mean levels of total and LDL cholesterol and triglycerides are considerably higher than the previous report in the general Chinese adult population [16–19]. Moreover, 56.02% of the Shandong general population aged ≥40 years have a borderline-high or high total cholesterol level, 38.63% have a borderline-high or high LDL cholesterol level, and 33.54% have a borderline-high or high triglycerides level.

Furthermore, glucose metabolism disorder leads to dyslipidemia. The total and LDL cholesterol and triglyceride levels increased by 4.17% (0.22), 5.14% (0.16), and 11.69% (0.18 mmol/L) in the population with prediabetes; and 2.28% (0.12), 3.86% (0.12), and 29.87% (0.46 mmol/L), respectively, in the population with diabetes, compared with those in the NGT population. In addition, HDL cholesterol level decreased by 2.60% (0.04) and 7.14% (or 0.11 mmol/L) in the populations with prediabetes and diabetes, compared with that in the NGT population. Thus, glucose metabolism disorder leads to the increase of the mean levels of serum total and LDL cholesterol and triglycerides, whereas the mean level of HDL cholesterol decreases. Additionally, glucose metabolism disorder greatly influences the triglyceride levels.

Several previous studies reported serum lipid and lipoprotein levels in Chinese population [16–19]. The China National Diabetes and Metabolic Disorders Study, which included 47,325 residents aged ≥20 years around China, showed that the mean serum total, LDL, and HDL cholesterol and triglyceride levels were 4.72, 2.68, 1.30, and 1.57 mmol/L, respectively [19]. In our study, which was only conducted 4–5 years later, the total and LDL cholesterol and triglyceride levels increased by 13.35% (0.63), 18.66% (0.50), and 7.01% (0.11 mmol/L), respectively. These increases are greatly evident and unlikely due to changes in laboratory measurement methods and different sampling methods. The serum lipid and lipoproteins are known to be closely related to age, and our study residents, which were aged ≥40 years, and those in previous studies have different age range. These facts may be the reason behind the different results acquired between our study and previous studies. Nevertheless, this distinction could only lead to minimal difference. Therefore, the apparent increase in serum total and LDL cholesterol and triglyceride levels is most likely true. Furthermore, it will continue to increase if without effective intervention.

These study results have immensely important public health implications. Traditionally, the mortality from atherosclerotic cardiovascular diseases has been uncommon in China and is estimated to be approximately one tenth of that in Western countries [20–23]. In our study, only 3% of the residents with dyslipidemia used lipid-lowering medication regularly. Without a national effective prevention and control of hypercholesterolemia, cardiovascular and cerebrovascular disease cases will continue to grow in China. However, contrary to the current situation in China [24–28], the serum cholesterol level in most countries worldwide has decreased remarkably over the past few decades [29–32].

The present study has some limitations. First, our study was conducted in Shandong Province; therefore, the estimated results could not be applied to the entire Chinese population. Shandong Province is one of the Chinese coastal provinces and has a higher level of economic development, which possibly explains the higher increase in the mean levels of serum lipids and lipoproteins in Shandong Province adults than that in the entire Chinese population. Second, the gender distribution is imbalanced. Females were overrepresented.

## Conclusion

Serum total and LDL cholesterol levels were high in the population aged ≥40 years of Shandong province. Age, gender, status of glucose metabolism, BMI, and HbA1c can affect serum lipids and lipoproteins. Without effective prevention and intervention, cardiovascular and cerebrovascular disease cases will continue to grow in the future in China.

## Abbreviations

ALT: Alanine aminotransferase
AST: Aspartate aminotransferase
BMI: Body mass index
Cr: Creatinine
FINS: Fasting plasma insulin
FPG: Fasting plasma glucose
HbA1c: Glycosylated hemoglobin
HDL: High-density lipoprotein
HOMA-IR: Homeostasis model assessment insulin resistance
IFG: Impaired fasting glucose
IGT: Impaired glucose tolerance
LDL: Low-density lipoprotein
NGT: Normal glucose tolerance
WHR: Waist-hip ratio
